# Genotypic variations in leaf and whole-plant water use efficiencies are closely related in bread wheat genotypes under well-watered and water-limited conditions during grain filling

**DOI:** 10.1038/s41598-019-57116-0

**Published:** 2020-01-16

**Authors:** Alejandro del Pozo, Ana María Méndez-Espinoza, Sebastián Romero-Bravo, Miguel Garriga, Félix Estrada, Marta Alcaíno, Anyela V. Camargo-Rodriguez, Fiona M. K. Corke, John H. Doonan, Gustavo A. Lobos

**Affiliations:** 1 Centro de Mejoramiento Genético y Fenómica Vegetal, Facultad de Ciencias Agrarias, Universidad de Talca, Talca, Chile; 2 Facultad de Ciencias Agrarias y Forestales, Universidad Católica del Maule, Curicó, Chile; 3 National Plant Phenomics Centre (NPPC), IBERS, Aberystwyth University, Aberystwyth, SY23 3EA UK; 4 Present Address: The John Bingham Laboratory, National Institute of Agricultural Botany (NIAB), Cambridge, United Kingdom

**Keywords:** Plant stress responses, Environmental sciences

## Abstract

Wheat plants growing under Mediterranean rain-fed conditions are exposed to water deficit, particularly during the grain filling period, and this can lead to a strong reduction in grain yield (GY). This study examines the effects of water deficit after during the grain filling period on photosynthetic and water-use efficiencies at the leaf and whole-plant level for 14 bread wheat genotypes grown in pots under glasshouse conditions. Two glasshouse experiments were conducted, one in a conventional glasshouse at the Universidad de Talca, Chile (Experiment 1), and another at the National Plant Phenomics Centre (NPPC), Aberystwyth, UK (Experiment 2), in 2015. Plants were grown under well-watered (WW) and water-limited (WL) conditions during grain filling. The reductions in leaf water potential (Ψ), net CO_2_ assimilation (An) and stomatal conductance (gs) due to water deficit were 79, 35 and 55%, respectively, during grain filling but no significant differences were found among genotypes. However, chlorophyll fluorescence parameters (as determined on dark-adapted and illuminated leaves) and chlorophyll content (Chl) were significantly different among genotypes, but not between water conditions. Under both water conditions, An presented a positive and linear relationship with the effective photochemical quantum yield of Photosystem II (Y(II)) and the maximum rate of electron transport (ETRmax), and negative with the quantum yield of non-photochemical energy conversion in Photosystem II (Y(NPQ)). The relationship between An and Chl was positive and linear for both water conditions, but under WL conditions An tended to be lower at any Chl value. Both, instantaneous (An/E) and intrinsic (An/gs) water-use efficiencies at the leaf level exhibited a positive and linear relationship with plant water-use efficiency (WUEp = plant dry weight/water use). Carbon discrimination (Δ^13^C) in kernels presented a negative relationship with WUEp, at both WW and WL conditions, and a positive relationship with GY. Our results indicate that during grain filling wheat plants face limitations to the assimilation process due to natural senesce and water stress. The reduction in An and gs after anthesis in both water conditions was mainly due a decline in the chlorophyll content (non-stomatal limitation), whereas the observed differences between water conditions were mainly due to a stomatal limitation.

## Introduction

In Mediterranean climatic regions, annual average temperatures have increased and precipitation decreased during the last century^1–3^, and this phenomenon has also affected Chile^4^. In addition, precipitation in Chile is strongly concentrated in winter and also manifests large inter-annual variability. Thus, wheat plants growing under rainfed conditions are exposed to a progressive water deficit starting from heading, leading to a ‘terminal drought stress’, which reduces grain yield (GY). Therefore, enhancing crop resilience is a priority for Mediterranean environments and may also provide a better understanding of the physiological mechanisms underlying water stress tolerance in wheat.

Under drought stress, net CO_2_ assimilation (An), stomatal conductance (gs) and transpiration (E) all decrease during grain filling, changing the instantaneous water-use efficiency (An/E) and intrinsic water-use efficiency (An/gs)^5–7^. At the plant level, water use efficiency (WUEp = DW/WU) corresponds to the coefficient between plant dry weight (DW) and the water-use (WU) by means of plant transpiration. According to Passioura 2006^8^, GY = WU × WUEp × HI, where HI is the harvest index (GY/DW), suggesting that under water stress conditions, the reduction in GY is a consequence of or at least correlated with lower WU and/or WUEp. If so, then the selection of genotypes with higher WU and/or WUEp should ameliorate the negative impact of water stress on GY. In a semi-controlled experiment, Guan *et al*.^7^ found a strong reduction in the WU and WUEp of wheat genotypes growing under water stress conditions, and significant genotypic differences for WU but not for WUEp. The above suggest a close relationship between the water use efficiency at leaf and plant level of wheat genotypes grown under different water conditions.

Terminal drought stress can also affect the properties and efficiency of the photosynthetic apparatus, particularly Photosystem II (PSII). Indeed, this is one of the first physiological processes affected, often prior to any other obvious stress symptoms^9^. Pulse amplitude modulated (PAM) fluorometry is a useful technique for identifying the amount of energy that is not used in photosynthesis but is re-emitted as fluorescence or dissipated as heat in the reaction centres, especially in PSII^10,11^. In particular, parameters from rapid light-response curves, which are quicker to obtain than the light response curves determined by infrared gas analysers, reveal key information about the rate of electrons travelling from PSII to PSI, and provide the photosynthetic photon flux density (PPFD) saturating the photosystems, and the efficiency with which electrons are used for photosynthesis^12,13^. There is little information about the relationship between An and parameters of chlorophyll fluorescence in wheat plants during grain filling under terminal drought stress.

Carbon isotope discrimination (Δ^13^C) in kernels represents another criterion for selecting high water-use efficiency genotypes^14^ and also provides an indirect determination of the effective water used by the crop^15,16^. Indeed, negative relationships between Δ^13^C and An/gs have been reported in wheat and other C3 crops^17–19^.

The aims of this study were a) to evaluate the performance of the leaf photosynthetic apparatus; and b) to evaluate the relationships between water-use efficiencies at the leaf and whole-plant level, and the Δ^13^C in kernels in14 wheat genotypes with different tolerance in terms of yield performance under terminal drought stress, in two contrasting water regimes during grain filling under glasshouse conditions. In a previous study we evaluated a large collection of 384 advanced wheat lines grown under water-limited and well-watered conditions^20^. We found large genotypic variability in grain yield under water-limited conditions, which was positively correlated with flag leaf chlorophyll content at anthesis and carbon discrimination in kernels, suggesting that cultivars that maintained higher levels of photosynthesis and conductance during grain growth were more productive. Also, the yield decrease under water-limited conditions during grain growth is related to various physiological changes including WU and water-use efficiency at the leaf and whole-plant level.

This characterisation should improve our understanding of genotype-dependent variation in water-use efficiency and its relationships with leaf water potential, leaf gas exchange, chlorophyll fluorescence and carbon isotope composition measurements (reverse phenomics), thus generating relevant information to be used for genotype selection (forward phenomics) under field conditions.

## Results

### Soil water content, leaf water potential and gas exchange

In experiment 1, the soil water content was maintained at an average of 0.168 and 0.06 m^3^ m^−3^ for well-watered (WW) and water-limited (WL) conditions, respectively, from fully expanded flag leaves (8 September) until maturity (Supplementary Fig. 1). Under WL conditions, leaf water potential (Ψ), An, gs and E at anthesis (Z65) were reduced by 32, 18, 38 and 30%, respectively, whereas at the hard dough grain stage (Z87) these were 79, 35, 55 and 50%, respectively. However, no significant differences were found among genotypes (Table 1). The instantaneous and intrinsic WUE significantly increased under WL, with the interaction between genotype × water condition (GxW) being highly significant (Table 1). All these traits exhibited a strong and significant reduction during grain filling, and in the case of Ψ and internal CO_2_ concentration (Ci), the water condition × phenology (WxP) interaction was significant (Table 1).

**Table 1:** Mean values (N = 56) of leaf water potential, leaf gas exchange, chlorophyll fluorescence parameters and chlorophyll content determined at anthesis (A) and grain filling (soft dough-SD and hard dough-HD grain), of 14 genotypes growing in a glasshouse under well-watered (WW) and water-limited (WL) conditions (Experiment 1). G – genotype; W – water regime; P – phenology.

Trait^a^	WW			WL			ANOVA			
	A	SD	HD	A	SD	HD	G	W	P	Interactions
Ψ (Mpa)	−0.55	−0.58	−1.01	−0.73	−0.93	−1.82	n.s.	***	***	WxP
An (μmol CO_2_ m^−2^ s^−1^)	21.1	14.7	7.6	17.2	12.0	4.9	n.s.	***	***	n.s.
gs (mmol H_2_0 m^−2^ s^−1^)	527.7	430.5	327.3	327.2	245.3	146.9	n.s.	***	***	n.s.
E (mmol H_2_0 m^−2^ s^−1^)	6.4	5.7	4.0	4.5	3.8	2.0	*	***	***	n.s.
Ci (mmol CO_2_ mol^−1^)	271.7	287.4	320.5	252.2	267.1	311.3	***	***	***	GxW, WxP
An/E (μmol CO_2_ mmol H_2_0^−1^)	3.4	2.7	1.9	3.9	3.3	2.3	***	***	***	GxW, GxP
An/gs (μmol CO_2_ mol H_2_0^−1^)	43.0	38.9	24.2	58.0	53.8	33.9	***	***	***	GxW
F_0_ (mv)	1.55	1.72	1.74	1.54	1.69	1.64	***	***	***	GxP, WxP
Fm (mv)	6.89	6.90	6.47	6.90	6.90	6.36	***	n.s	***	GxP
Fv/Fm	0.80	0.76	0.72	0.80	0.76	0.75	***	n.s.	***	GxP
∼F_0_’ (mv)	1.39	1.46	1.30	1.37	1.45	1.23	***	**	***	GxP
Fm’ (mv)	3.35	3.58	2.64	3.33	3.60	2.54	***	n.s.	***	GxP
Alpha (e^−^/ photons)	0.37	0.37	0.41	0.36	0.38	0.40	n.s.	n.s.	***	n.s.
IK (μmol photons m^−2^ s^−1^)	286.7	195.9	137.8	296.5	208.8	150.4	n.s.	n.s.	***	n.s.
ETRmax (μmol e^−^ m^−2^ s^−1^)	99.6	74.8	55.9	102.7	75.8	59.6	n.s.	n.s.	***	n.s.
Y(II)	0.16	0.13	0.10	0.16	0.13	0.09	*	n.s.	***	n.s.
Y(NPQ)	0.43	0.42	0.55	0.44	0.42	0.54	***	n.s.	***	n.s.
Y(NO)	0.41	0.46	0.38	0.41	0.46	0.37	n.s.	n.s.	***	GxP
Chl (Dualex units)	46.69	42.48	12.86	46.81	44.12	13.12	***	n.s	***	GxW, GxP

^a^Ψ: leaf water potential; An: leaf net CO_2_ assimilation; gs: stomatal conductance; E: transpiration rate; Ci: internal CO_2_ concentration; An/E: instantaneous water use efficiency; An/gs: intrinsic water use efficiency; F_0_ and Fm: minimum and maximum fluorescence in the dark-adapted state, respectively; Fv/Fm: maximum photochemical quantum yield of PSII; ∼F_0_’ and Fm’: respectively the calculated minimum and maximum chlorophyll fluorescence yield in PSII reaction centres in the open state; Alpha: initial slope of the light curve, related to maximum yield of photosynthesis; IK: PAR value of the point of intersection between the horizontal line ETRmax and the extrapolated initial slope; ETRmax: maximum rate of electron transport; Y(II) = (Fm’-F)/Fm’): effective photochemical quantum yield of photosystem II; Y(NPQ): quantum yield of non-photochemical energy conversion in PS II due to down-regulation of the light-harvesting function; Y(NO): quantum yield of non-photochemical energy conversion in PS II other than that caused by down-regulation of the light-harvesting function; Chl: chlorophyll content. Significance levels: *(P < 0.05), **(P < 0.01), ***(P < 0.001), n.s. (differences not significant; P > 0.05).

In experiment 2, the WL condition led to a strong reductions in An, gs and E at heading of 26, 44 and 40%, respectively, but no significant differences were detected among the six genotypes (Table 2). The instantaneous and intrinsic WUE increased under WL conditions by 23 and 33%, respectively. The GxW interaction was not significant for any of these traits (Table 2).

**Table 2:** Mean values (n = 30) of leaf gas exchange, chlorophyll fluorescence parameters and chlorophyll content determined at heading in six genotypes growing in a LemnaTec glasshouse under well-watered (WW) and water-limited (WL) conditions (Experiment 2). G – genotype; W – water regime.

Trait^a^	Water condition		ANOVA		
	WW	WL	G	W	GxW
An (μmol CO_2_ m^−2^ s^−1^)	14.60	10.83	n.s.	**	n.s.
gs (mmol H_2_0 m^−2^ s^−1^)	172.01	96.05	n.s.	***	n.s.
E (mmol H_2_0 m^−2^ s^−1^)	1.88	1.13	n.s.	***	n.s.
Ci (mmol CO_2_ mol^−1^)	246.6	203.69	n.s.	***	n.s.
An/E (μmol CO_2_ mmol H_2_0^−1^)	7.93	9.77	n.s.	***	n.s.
An/gs (μmol CO_2_ mol H_2_0^−1^)	89.06	118.1	n.s.	***	n.s.
ETR (μmol e^-^ m^−2^ s^−1^)	96.69	88.49	*	n.s.	n.s.
Y(II)	0.15	0.14	*	n.s.	n.s.
Chl (SPAD units)	45.75	46.7	***	n.s.	n.s.

^a^Abbreviations are as in Table 2. Significance levels: *(P < 0.05), **(P < 0.01), ***(P < 0.001), n.s. (differences not significant; P > 0.05).

### Chlorophyll fluorescence and content

The minimum (Fo) and maximum (Fm) fluorescence in the dark-adapted state, and the maximum photochemical quantum yield of PSII (Fv/Fm) were significantly different among the 14 genotypes (experiment 1), but between water conditions and the GxW interaction there were only significant differences for Fo; the genotype × phenology interaction was significant for the three parameters (Table 1). For the chlorophyll parameters determined under illuminated conditions, genotypic differences were detected for the minimum (∼Fo’) and maximum (Fm’) chlorophyll fluorescence yield, effective photochemical quantum yield of PSII [Y(II)] and quantum yield of non-photochemical energy conversion in PSII due to down-regulation of the light-harvesting function [Y(NPQ)], but between the water conditions there were only significant differences for ∼Fo’ (Table 1). The chlorophyll parameters changed significantly with the phenological stage and the WxP interaction was significant in the case of Fo, Fm, Fv/Fm, ∼Fo’, Fm’ and the quantum yield of non-photochemical energy conversion in PSII other than that caused by down-regulation of the light-harvesting function [Y(NO)] (Table 1). In Experiment 2, significant genotypic differences were observed for the maximum rate of electron transport (ETRmax) and Y(II), but there were no differences between water conditions or GxW interaction (Table 2).

The chlorophyll content was also significantly different among the 14 genotypes, as well as the genotype × water condition interaction (Table 1). Similarly in experiment 2, the chlorophyll content was different among genotypes, but the GxW interaction was not significant (Table 2). No significant differences in Chl content were detected between water conditions in either experiment.

### Relationships between leaf water potential, leaf gas exchange and chlorophyll fluorescence

**Figure 1:**
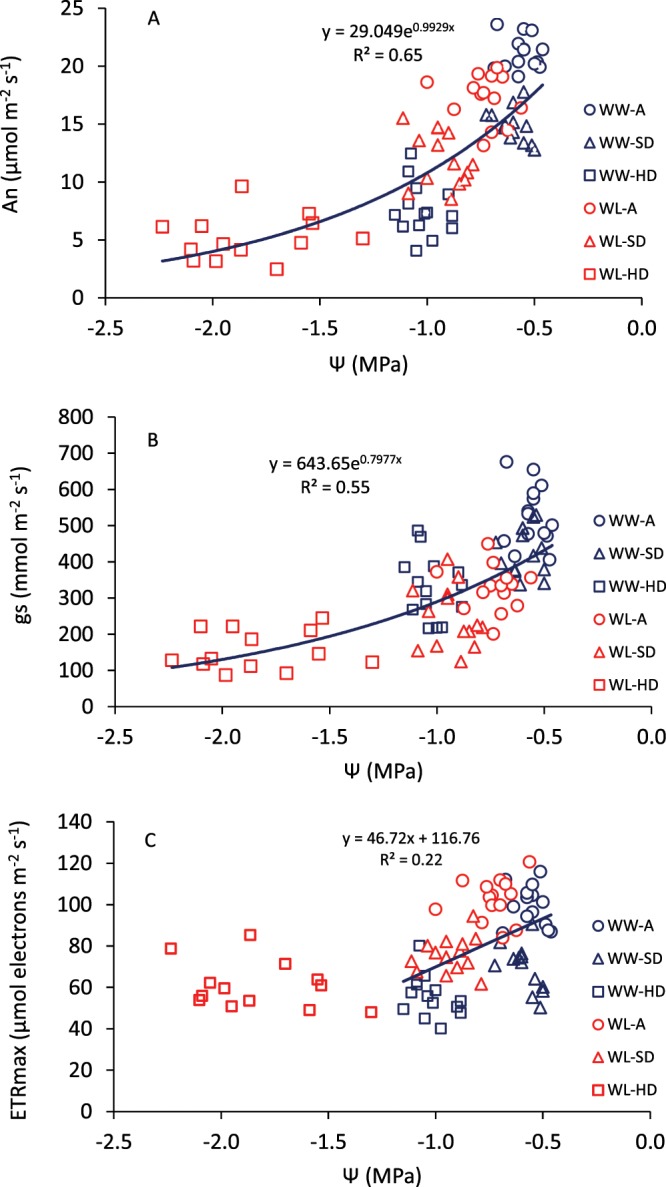
Relationships between leaf water potential and (**A**) net CO_2_ assimilation at light saturation (An), (**B**) stomatal conductance (gs) and (**C**) maximum rate of electron transport (ETRmax), determined at anthesis (**A**) and grain filling (soft dough-SD and hard dough-HD grain) of 14 genotypes of wheat grown under well-watered (WW) and water-limited (WL) conditions in a glasshouse. Values are the means of four replicate plants.

Both An and gs decreased exponentially as the Ψ declined from −0.5 to −2.2 MPa, with no apparent differences in the response between water conditions. At Ψ < −1.2 MPa, the leaves were under severe water stress, with An and gs reaching 5 µmol m^−2^ s^−1^ and 100 mmol m^−2^ s^−1^, respectively (Fig. 1A,B). In the case of ETRmax, the reduction occurred up to Ψ = −1.2 MPa and below that, ETRmax was on average 60 µmol e^-^ m^−2^ s^−1^ (Fig. 1C).

**Figure 2:**
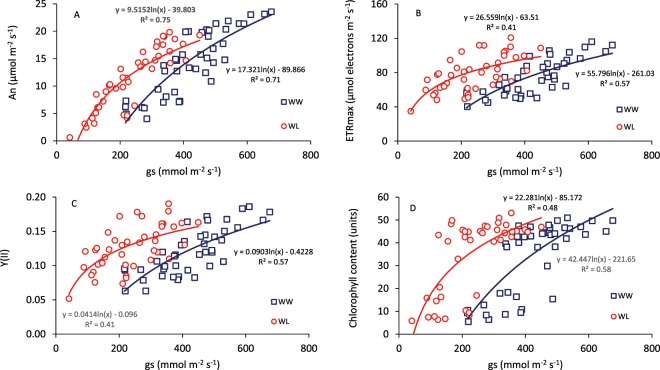
Relationship between stomatal conductance (gs) and (**A**) net CO_2_ assimilation at light saturation (An), (**B**) maximum electron transport (ETRmax), (**C**) effective photochemical quantum yield of photosystem II, and (**D**) the chlorophyll index (Dualex), determined at anthesis and grain filling in 14 genotypes of wheat grown in a glasshouse under well-watered (WW) and water-limited (WL) conditions. Each data point is the mean of four replicates.

Positive and curvilinear relationships were found between gs and An, ETRmax, Y(II) and the chlorophyll index (Chl), under WW and WL conditions (Fig. 2). In general, plants growing under WL exhibited higher levels of An, ETRmax, Y(II) and Chl compared to WW conditions, at any value of gs.

**Figure 3:**
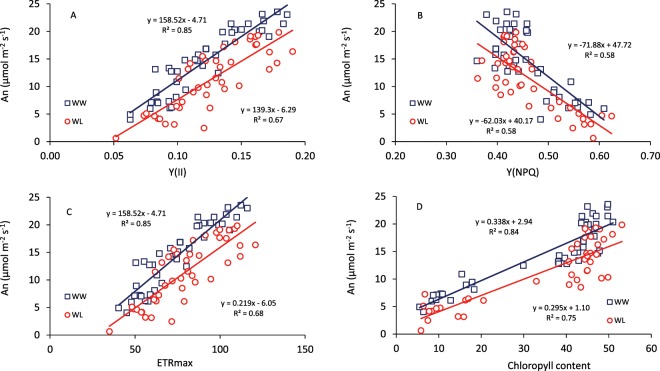
Relationship between leaf photosynthesis at light saturation (An) and (**A**) effective photochemical quantum yield of photosystem II, (**B**) quantum yield of non-photochemical energy conversion in PS II (Y(NPQ), (**C**) maximum electron transport (ETRmax) and (**D**) the chlorophyll index (Dualex), determined at anthesis and grain filling in 14 genotypes of wheat grown in a glasshouse under well-watered (WW) and water-limited (WL) conditions. Each data point is the mean of four replicates.

Under both water conditions, An presented a positive and linear relationship with Y(II) and ETRmax, and a negative relationship with Y(NPQ) (Fig. 3A–C); under WL, slopes and intercepts of the linear relationships were slightly lower compared to WW conditions. The relationship between An and Chl was positive and linear for both water conditions, but under WL conditions An tended to be lower at any Chl value (Fig. 3D).

### Green area, plant dry weight, dry matter partitioning, grain yield and yield components

**Figure 4:**
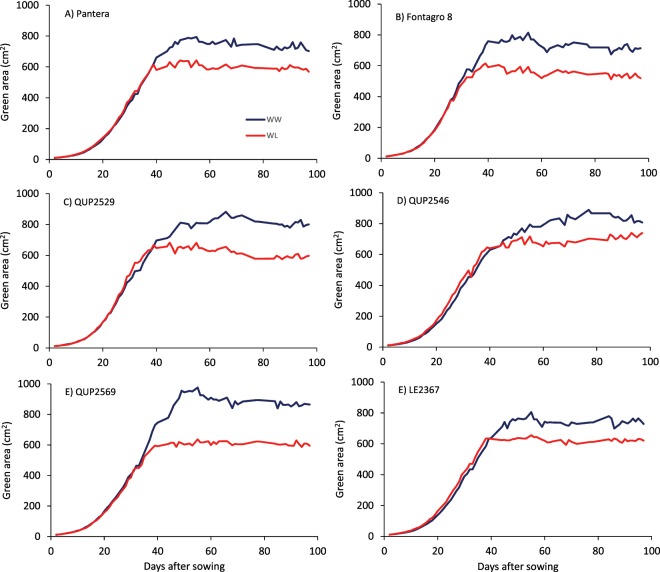
Average values (n = 5) of green area calculated for six genotypes of spring wheat grown in a LemnaTec glasshouse under well-watered (WW) and water-limited (WL) conditions in 2015 (Experiment 2). The beginning of the two water regimes was 31 days after sowing when most of the genotypes presented fully expanded flag leaves (Z41).

The green area of the six genotypes (experiment 2) showed significant genotypic differences and large differences between water conditions (Fig. 4; Table 3). Under WL conditions, the green area declined soon after the water treatment started at 31 days after sowing when most genotypes presented fully expanded flag leaves (Z41). At 96 days after sowing, the average reduction in green area as a consequence of water deficit was 23.7%. The strongest reduction in green area under WL conditions was observed in genotype QUP2569 and the lowest in QUP2546.

**Table 3:** Mean values of green area, plant dry weight, spike weight, plant water use (WU) and plant water-use efficiency (WUE) of six genotypes growing in a LemnaTec glasshouse under well-watered (WW) and water-limited (WL) conditions (Experiment 2). G – genotype; W – water regime.

Trait	Water condition		ANOVA		
	WW	WL	G	W	GxW
Green area (cm^−2^)	797.2	608.6	***	***	n.s.
Plant dry weight (g)	51.5	33.3	*	***	*
Spike weight (g)	29.5	18.8	n.s.	***	n.s.
Plant water use (L plant¯¹)	16.1	9.6	*	***	n.s.
Plant WUE (g L^−1^)	3.2	3.5	n.s	***	*

Significance levels: *(P < 0.05), **(P < 0.01), ***(P < 0.001), n.s. (differences not significant; P > 0.05).

DW, GY, number of spikes per plant (SP), number of kernels per spike (KS) and per plant (KP), the thousand kernel weight (TKW), the root:shoot ratio (R:S), and the harvest index (HI) in experiment 1 exhibited significant differences among genotypes (Table 4). The effect of water condition was also significant except for KS, HI and R:S (Table 4). The average HI was high (>0.50) but there was one genotype (QUP2546-2009) that exhibited the lowest HI (0.38) in both water conditions. Under WL conditions, DW, GY and KP were reduced by about 25%, while TKW increased by 4%. The GxW interaction was not significant (P > 0.05) for any trait (Table 4). GY for the 14 genotypes presented positive and significant (P < 0.001; n = 56) correlations with SP (r = 0.64 and 0.48 for WW and WL, respectively), KP (r = 0.71 and 0.59) and HI (r = 0.54 and 0.72). In experiment 2, DW was significantly different (P < 0.05) among genotypes, as was the GxW interaction (Table 3). The WL condition reduced DW by 35% and the spike weight by 36% (Table 3).

**Table 4:** Mean values of plant dry weight, grain yield and its agronomic components, dry matter partitioning, apparent water use, plant water-use efficiency (WUE) and carbon isotope discrimination in kernels (Δ^13^C), of 14 genotypes growing in a glasshouse under well-watered (WW) and water-limited (WL) conditions (Experiment 1). G – genotype; W – water regime.

Trait	Water condition		ANOVA		
	WW	WL	G	W	GxW
Plant dry weight (g)	35.3	26.5	***	***	n.s.
Grain yield (GY; g plant^−1^)	17.0	13.0	***	***	n.s.
Spikes per plant	6.4	5.0	***	*	n.s.
Kernel per spike	51.7	49.6	**	n.s.	n.s.
Thousand kernel weight (g)	51.8	53.8	***	***	n.s.
Kernel per plant	330.5	248.4	***	***	n.s.
Harvest index	0.52	0.53	***	n.s.	n.s.
Root:shoot	0.10	0.09	*	n.s.	n.s.
Plant water use (WU; L plant^−1^)	7.3	4.9	**	***	n.s.
Plant WUE (g L^−1^)	4.9	5.5	***	***	n.s.
GY/WU (g L^−1^)	2.4	2.7	***	***	n.s.
Δ^13^C in kernel (‰)	20.31	19.02	**	***	n.s.

Significance levels: *(P < 0.05), **(P < 0.01), ***(P < 0.001), n.s. (differences not significant; P > 0.05).

### Plant water-use, water-use efficiency and carbon isotope discrimination in grain

The WU per plant was reduced under WL conditions after the start of the water treatment (Supplementary Fig. 2). The total WU during the whole growing period was significantly different (P < 0.01) among genotypes and between water conditions, but the GxW interaction was not significant in either experiment. Under WL conditions, the average plant WU decreased by 33 and 40% in experiments 1 and 2, respectively (Tables 3 and 4).

**Figure 5:**
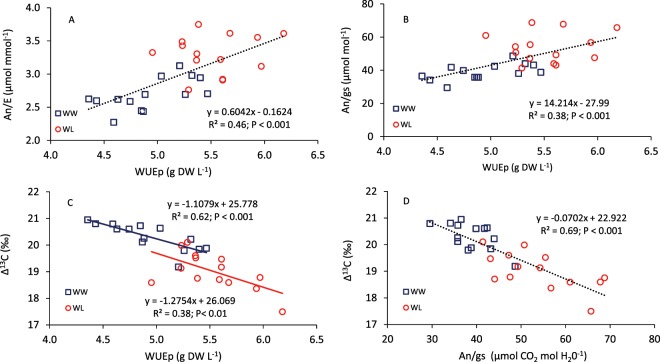
Relationships between plant water use efficiency (WUEp) and (**A**) instantaneous water use efficiency (An/E), (**B**) intrinsic water use efficiency (An/gs) and (**C**) carbon isotope discrimination in kernels (Δ^13^C, ‰), and (**D**) between An/gs and Δ^13^C, of 14 genotypes of wheat grown under well-watered (WW) and water-limited (WL) conditions in a glasshouse. Data for An/E and An/gs were measured at grain filling (soft dough grain) and values are the means of four replicate plants.

WUEp under WL conditions increased by 12% and 9% in experiments 1 and 2, respectively (Tables 3 and 4). Differences among water conditions were significant (P < 0.001) in both experiments, but the GxW interaction was only significant (P < 0.05) in experiment 2. The water productivity (GY/WU) under WL conditions was also increased by 13% in experiment 1, and was significantly different (P < 0.001) among genotypes (Table 4). Δ^13^C was also different among genotypes (P < 0.01), and was significantly higher (P < 0.001) under WW conditions (Table 4). WUEp determined under WW and WL conditions presented a positive and linear relationship with the instantaneous (An/E) and intrinsic (An/gs) water-use efficiencies evaluated at grain filling (Fig. 5A,B) or at anthesis (not shown), but had a negative relationship with Δ^13^C (Fig. 5C). In addition, Δ^13^C showed a negative and linear relationship with An/gs (Fig. 5D) and a positive correlation with GY (r = 0.6; P < 0.001, n = 28).

## Discussion

### Agronomic traits

The 14 advanced wheat lines and cultivars evaluated in this work exhibited a high phenotypic variability for leaf and whole-plant traits. The genotypes have a very different genetic background except for cvs. Pantera-INIA and Pandora-INIA, which are very similar because Pantera-INIA is a Clearfield® cultivar with resistance to the herbicide imidazolinone following introduction of the Ser-Asn627 mutation into two acetolactate synthase (ALS) genes (imi1 and imi2), located in wheat on chromosomes 6B and 6D, respectively, into cv. Pandora-INIA, by a biotechnological procedure^21^. In experiment 1, the average DW, GY and HI of Pandora-INIA and Pantera-INIA were not significantly different (P < 0.05), however, field experiments conducted in different environments showed consistently high GY in Pantera-INIA (http://www.semillasinia.cl/wp-content/uploads/2012/09/FolletoPanteraINIA.pdf ^20^;), indicating that the two cultivars probably respond differently to agronomic management.

The strong correlation between HI and GY (r = 0.72; P < 0.001) under WL conditions suggests that the partitioning of the aboveground biomass to the grain is an important trait for selecting genotypes for high-yielding and drought-prone environments where water deficit occurs mainly during grain filling. Retrospective studies of old and modern cultivars in Mediterranean environments have shown that progress in breeding for yield after 1900 has been strongly associated with increases in HI in bread^15,22^ and durum wheat^23^.

### Photosynthetic traits

The strong decline in An, gs and E from anthesis to the hard dough grain stage under both water conditions (Table 1) was associated with a decrease in Ψ and Chl content during grain growth (Fig. 1). However, no genotypic differences in A and gs were detected among genotypes (Tables 1 and 2), probably because all the material was modern and possessed high yield potential under well-watered conditions (Table 5). Other studies comparing wheat cultivars (from different years of release) under well-watered conditions have reported an increase of gs in modern cultivars in close relationship with GY; for instance, in Mexico^24^, China^25^ and Chile^22^. A recent study conducted on 64 field-grown wheats in the UK demonstrated that photosynthetic traits (leaf gas exchange and chlorophyll a fluorescence) were positively correlated with GY and harvest index^26^. During grain filling in the current work, both An and Chl decreased as a consequence of the process of leaf senescence, explaining the positive correlation between both traits (Fig. 3D). However, the Chl content of flag leaves was similar in both water conditions (Tables 1 and 2). In addition, the Chl content exhibited significant genotype × phenology and genotype × water condition interactions (Table 1), suggesting that some genotypes may present a more delayed senescence (stay-green). There is evidence that the stay-green phenotype or plants with delayed leaf senescence can improve their performance under drought conditions^27,28^. For example, durum wheat (*Triticum turgidum* ssp. *durum*) mutants growing under glasshouse conditions remained green for longer and had higher rates of leaf photosynthesis and larger seed weight^29^. Other studies conducted in field conditions reported a positive correlation between the stay-green trait and GY^30,31^. This capacity for maintaining photosynthetic rates under stress conditions is related to the capacity of stay-green genotypes to have low chlorophyll a/b ratios and more chlorophyll a and b content than genotypes without this characteristic^32^. Therefore, this ideotype also shows high values of parameters associated with efficient conversion of photosynthetic energy, such as Y(II) or ETRmax^33^.

**Table 5:** Selected genotypes, grain yield (GY) and the yield tolerance index (YTI) determined at two Mediterranean sites, Cauquenes under water stress (WS) and Santa Rosa under full irrigation (FI) conditions, in 2012. Experiment 1 and 2 refers to the trial conducted in a conventional glasshouse at the Plant Breeding and Phenomic Center, Talca, Chile, and in a LemnaTec glasshouse at the National Plant Phenomics Centre (NPPC), Aberystwyth, UK, respectively. The genotypes were selected from a set of 384 genotypes (del Pozo *et al*.^20^.

Genotype name	Pedigree	GY_WS_	GY_FI_	YTI^*^	Experiment
		(Mg ha^−1^)	(Mg ha^−1^)		
QUP2418-2007	ALTAR84/AE.SQUA (221)//SIREM/3/SRMA/TUI	5.5	12.0	0.67	1
QUP2546-2009	MILAN/PASTOR//DOMO	5.4	10.2	0.56	1 and 2
FONTAGRO 8	KA/NAC//SERI/RAYON	5.0	10.2	0.52	1 and 2
LE 2367	LE2265/LE2304	4.9	9.5	0.47	1 and 2
QUP2529-2009	RL6043/4*NAC//QUP 1861_96	4.3	10.1	0.44	1 and 2
QUP2474-2007	SITE//BUC/PVN/3/QUELEN	4.0	10.8	0.44	1
PANTERA-INIA	TJB358.251/BUC//CIKO	3.4	11.0	0.38	1 and 2
FONTAGRO 92	PFAU/BOW//VEE#9/3/WBLL1	4.1	9.0	0.37	1
QUP2405-2006	QUP 1865_96/CAR3911//QUP 1865_96	3.8	9.4	0.36	1
QUP2616-2009	PFAU/WEAVER*2//PAVON 7S3, + LR47	3.2	10.3	0.33	1
LE 2384	BAG10/B. Sureño	3.4	9.1	0.31	1
PANDORA-INIA	TJB358.251/BUC//CIKO	2.8	9.3	0.26	1
QUP2569-2009	MILAN/PASTOR//DOMO	2.0	10.2	0.21	1 and 2
FONTAGRO 98	FILIN/IRENA/5/CNDO/R143//ENTE/MEXI_2/3/AEGILOPS SQUARROSA(TAUS) /4/WEAVER/6/BERKUT	1.6	9.2	0.15	1

^*^YTI = Y_WS_Y_FI_/Ῡ_FI_^2^ where Y_WS_ and Y_FI_ are the genotype yield under water stress (Cauquenes) and full irrigation conditions (Santa Rosa), respectively, and Ῡ_FI_ is the mean yield of all genotypes under FI conditions; the higher the YTI value the better the performance of the genotype under rainfed conditions.

Chlorophyll fluorescence parameters determined in dark-adapted leaves (Fo, Fm and Fv/Fm) and light-adapted leaves (∼Fo’, Fm’, Y(II) and Y(NPQ)) exhibited significant differences among genotypes (Tables 1 and 2). For example, Pantera-INIA presented 10% higher values for Y(NPQ) and 9% lower for Y(NO) at anthesis and grain filling, under WL conditions, and this may explain the better performance of Pantera-INIA in rainfed conditions. A study of 144 genotypes of durum wheat, cultivated in three Mediterranean conditions, demonstrated that Fo and Fm had a strong genotypic effect and also exhibited high broad-sense heritabilities^34^. Also, the same authors reported that Fo, Fm and Fv/Fm were significantly correlated with GY.

Fv/Fm and Y(II) are widely used as indicators of the degree plant stress and as criteria for genotype selection, in drought or heat ambient^35–37^. Decreases in both parameters are associated with greater heat release in the antenna complex and the reduction in the amount of light energy used for photosynthesis increases photo-oxidative damage^38,39^. In this work Fm’, Fv/Fm and Y(II) decreased during grain filling under WW and WL conditions, but no differences were detected between the two water conditions (Table 1), indicating that PSII was not damaged during the water stress, and the decline in An was mainly due to stomatal limitation^40^. In addition, changes in gs and An during grain filling were closely related to changes in ETRmax and Y(II) (Figs. 2 and 3). It has been suggested that a decrease in Rubisco activity in C3 plants typically occurs at lower gs < 100 mmol H_2_0 m^−2^ s^−1^, whereas permanent biochemical limitations are observed at very low gs (<50 mmol H_2_0 m^−2^ s^−1^ ^41^. Under water deficit conditions, it was observed that at gs <~100 mmol H_2_0 m^−2^ s^−1^, which occurred at the hard dough grain stage, there was a strong decline in An, ETRmax and Y(II) (Fig. 3), indicating non-stomatal limitations^41^. Under WW conditions, the non-stomatal limitations started at higher gs compared with WL conditions, and this could have been a consequence of the leaf senescence process.

The decrease or loss of photosynthetic capacity in plants is an inherent process of senescence, which is more pronounced under water deficit. However, biochemical limitations appear mainly in late stages of grain filling or under severe water deficit^42–44^. As the flag leaf senesced (in either water condition), the chlorophyll content and the Fm’ values declined (Table 1), which indicates that the PSII centres were partially closed (Quinone A reduced) and that the excess energy dissipated mainly via heat emission^9^. Thus, the performance of the photosynthetic process during grain growth was affected by stomatal and non-stomatal limitations^40,45,46^.

### Leaf and whole-plant water-use efficiencies, and carbon discrimination

When water deficits start to build up, both leaf water potential and stomatal conductance decrease faster than carbon assimilation, leading to an increase in instantaneous and intrinsic water-use efficiency^47^. The relationship between gs and An in flag leaves evaluated at anthesis and grain filling (Fig. 2) indicated that An/gs was higher under WL conditions in all genotypes. It seems that an acclimation process occurred at the leaf level when wheat plants were exposed to terminal water stress, leading to a better use of the available water. The genotypic differences in An/gs and An/E, and also the significant GxW interaction found at anthesis and grain filling (Tables 1 and 2) suggests that genotypes have different sensitivity to the water deficit. In a comparison of six wheat genotypes Skider *et al*.^6^ also found a strong reduction in An/E and An/gs under water deficit conditions, and reported a significant GxW interaction.

The water deficit during grain filling (terminal drought) caused a strong and rapid reduction in the green area (Fig. 4), as well as the WU and DW (Tables 3 and 4). Because the terminal drought produced a stronger reduction in WU (33 and 40% in experiments 1 and 2, respectively) compared to the observations for DW (25 and 35%, respectively), there was an increase in WUEp under WL conditions. This was also observed in the water-use efficiency evaluated at the leaf level (An/E and An/gs). Indeed, the genotypic variability in WUEp under WW and WL conditions was closely related to the variability observed in An/E and An/gs (Fig. 5). Here, the scaling up from single leaf to the whole plant revealed significant correlations, but this is not always the case, particularly in field-grown plants^48^. Indeed, a positive and significant correlation (r = 0.66; P = 0.014, N = 13) was observed between the GY of the genotypes in Mediterranean rainfed (water stress) conditions and the water productivity (GY/WU) determined under WL conditions in experiment 1, when the genotype with the lowest HI (QUP2546-2009) was excluded from the analysis.

The above observations raise the question of which types of genotypes should be selected for Mediterranean conditions. Selecting for higher WUEp has usually led to drought-tolerant genotypes with lower WU and biomass and, therefore, reduced grain yield. Perhaps our target should be genotypes with higher biomass and HI under water-limiting conditions, although this implies higher WU.

Carbon discrimination in kernels was negatively correlated to WUEp and An/gs (Fig. 5), as reported by other authors in wheat genotypes^19,49^, and positively correlated with GY. In fact, the Δ^13^C in kernels can be positive or negatively correlated with GY depending on soil water availability^20,50–52^. In Mediterranean conditions, negative relationships between GY and δ^13^C (or positive with Δ^13^C) have been frequently reported in cereals^22,50,51,53^ and this can be explained as the genotypes exhibiting the highest water use also tending to be the most productive^16,51,54^. It also indicates that more productive genotypes are those maintaining stronger transpiration and thus increased water-use. Therefore, in wheat breeding programs oriented towards water-limiting conditions, efforts should focus on the identification of individuals with the capacity to generate greater biomass and HI, and with the associated higher WU and transpiration rates^16^.

## Conclusions

The 14 chosen spring bread wheat genotypes had contrasting GY under WL conditions and exhibited high phenotypic variability for WU and WUEp as evaluated in LemnaTec and conventional glasshouses. The WL conditions during grain filling (terminal drought) led to an abrupt interruption of green area development, a reduction in DW, and an improvement in WUE at the leaf and plant levels. The reduction in GY under WL conditions was associated with lower numbers of spikes per plant and kernels per plant, but not the TKW, which was slightly higher under WL conditions. It is possible that the lower number of kernels per plant reduced the competition from assimilates.

In Mediterranean environments, wheat plants experience natural senesce and water stress during grain filling, which both induce limitations on the assimilation process. The reduction in An and gs after anthesis under both water conditions was mainly due a reduction in the chlorophyll content (non-stomatal limitation), whereas the observed differences between water conditions were mainly due to stomatal limitation. The chlorophyll fluorescence parameters determined in dark-adapted leaves (Fo, Fm and Fv/Fm) and light-adapted leaves (∼Fo’, Fm’, Y(II) and Y(NPQ)) did not exhibit significant differences between water conditions, except for Fo and ∼Fo’. Indeed, the Chl content was similar in both water conditions, and this could explain the increase in WUE at the leaf level under water stress. The genotypic variabilities in An/E and An/gs were closely related to the variability observed in WUEp. Carbon discrimination (Δ^13^C) in kernels was negatively correlated with An/gs and positively correlated with GY.

## Materials and Methods

### Plant material, growing conditions and experimental design

Two experiments were conducted under glasshouse conditions. In Experiment 1, a set of 14 contrasting advanced spring bread wheat lines and cultivars with similar phenology were selected (according to their yield tolerance index (YTI) evaluated under field conditions (rain fed *vs*. full irrigation) from a previous study of 384 genotypes^20^ (Table 5). Experiment 1 was performed at the Plant Breeding and Phenomic Center (35° 24'19” S; 71° 37'59” W), Universidad de Talca, Talca, Chile, from 3 July to 13 November 2015. The glasshouse had natural lighting and a heating system; mean temperatures in the glasshouse ranged from 9–21 °C, and the daily average for the growing period was 16 °C (Supplementary Fig. 3). On 3 July 2015, ten seeds of each genotype were sown in 7.5 L pots of 26 cm in diameter and 21.1 cm height, filled with a 1: 1: 1 mixture of organic soil mixture (Anasac, Chile), perlite and river sand, representing a total weight per pot of 4.9 kg. After the emergence of the second leaf, the seedlings were thinned to five per pot. The soil moisture content of all pots was kept at 75% field capacity (FC) of the substrate until the flag leaf was fully expanded (Zadoks stage Z41)^55^ in most genotypes (8 September). To obtain the maximum water holding capacity the pots were watered until saturation and after 24 hours of free drainage were weighed to determine the amount of water required to reach 100% of FC (1,800 ml of water per pot). Therefore, 75% and 30% FC corresponded to 1,350 ml and 540 ml per pot, respectively. To maintain these two water conditions, each pot was weighed daily before and after each irrigation, and the water was replenished until the weights set in each treatment were reached. Additionally, soil water content (m^3^ m^−3^) was monitored using automatic sensors (EC-5, Decagon Devices Inc., WA, USA) installed at 5 cm depth and connected to a datalogger (EM-50, Decagon Devices Inc., WA, USA); there were three sensors per water condition (Supplementary Fig. 1). Micro- and macro-nutrient fertilisation was applied using Hoagland nutrient solution, at the rate of 200 ml per pot per week, from 15 July to 3 September 2015. The experimental design was completely randomised with two factors, genotype and water condition, and four replicates.

In Experiment 2, a subset of five contrasting advanced lines, four tolerant and one susceptible, and one cultivar (Table 5) were grown under LemnaTec glasshouse conditions at the National Plant Phenomics Centre (NPPC), Aberystwyth University, UK. Temperature conditions were kept between 18 and 20 °C. Natural lightning was supplemented with 600 W sodium lamps (~400 µmol m^−2^ s^−1^) on cloudy days. On 07 May 2015, two seeds per pot were sown in 1 L pots filled with Levington F2 compost. After germination the seedlings were thinned to one per pot. Plants were transferred to the glasshouse where each pot was placed into a cart on a conveyor system. Pots were weighed and watered automatically to 75% gravimetric water content daily. After the flag leaf was fully expanded (Z41; 10 June), a set of plants were kept at 75% field capacity (WW) and another at 30% FC (WL), giving a total of 60 plants. The experimental design was completely randomised with two factors, genotype and water condition, and five replicates.

### Leaf water potential, gas exchange, chlorophyll fluorescence and content

In Experiment 1, all measurements at leaf level were taken from the middle part of flag leaves of three plants per pot, under clear sky conditions and between 12 and 16 h. An average of three leaves was calculated for each replicate. Leaf water potential (Ψ) was determined using a pressure chamber (PMS 600, PMS Instrument Co., OR, USA). In order to equilibrate the potential of the leaf with the stem, flag leaves were covered with adhesive plastic film and aluminium foil for 2 h before measurements. Leaf net CO_2_ assimilation (An), stomatal conductance (gs), internal CO_2_ concentration (Ci) and transpiration rate (E) were determined on flag leaves at three phenological stages (P): anthesis (Z65) and soft (Z83) and hard dough grain (Z87), Zadoks *et al*.^55^, using a portable infra-red gas analyser (CIRAS 2, PP System, England). Measurements were performed using a cuvette of 1.7 cm^2^ of leaf area, at a flow rate of 250 ml min^−1^, reference CO_2_ of 380 µmol mol^−1^, leaf temperature of 25 °C and photosynthetic photon flux density (PPFD) of 1,500 µmol m^−2^ s^−1^. The instantaneous and intrinsic water-use efficiencies were calculated as An/E and An/gs, respectively.

PAM fluorescence was assessed with a portable fluorometer (PAM-2500, Walz, Germany) using the same leaves and phenological stages used to determine gas exchange. Minimum (Fo) and maximum (Fm) fluorescence in the dark-adapted state, and the maximum photochemical quantum yield of PSII (Fv/Fm) were assessed using leaf clips (DLC-8, Walz, Germany). Leaves were dark-acclimated for 20 min before measurements were taken. Next, each leaf clip was opened and, after at least 30 min of light acclimation, rapid light-response curves (RLCs) were determined using the amplitude modulated pulse mode; for each measurement the equipment provided 10 pulses every 6 s, which increased light from 0 µmol m^−2^ s^−1^ to 2,000 µmol m^−2^ s^−1^. After each pulse, the equipment delivered a saturating light pulse (10,000 µmol m^−2^ s^−1^), which allows the calculation of the minimum (~F_0_’) and maximum (Fm’) chlorophyll fluorescence yield during the open state of PSII reaction centres. In the RLC, at 1,500 µmol m^−2^ s^−1^, the following parameters were calculated: Y(II): effective photochemical quantum yield of PSII [(Fm’-F)/Fm’], where F is the fluorescence shortly before a saturating pulse; (Y(NPQ): quantum yield of non-photochemical energy conversion in PSII due to down-regulation of the light-harvesting function [(F/Fm’)-(F/Fm)]; and Y(NO): quantum yield of non-photochemical energy conversion in PSII other than that caused by down-regulation of the light-harvesting function [(F/Fm)]. After fitting the RLC, the following parameters were determined with the equations described by Eilers and Peeters 1988^56^: Alpha: initial slope of the light curve, related to the maximum yield of photosynthesis; ETRmax: maximum rate of electron transport; and IK: PAR value at the intersection of alpha and ETRmax. Following gas exchange and modulated chlorophyll fluorescence assessments, the chlorophyll content was estimated using a portable non-destructive chlorophyll meter (Dualex Scientific, Force-A).

In Experiment 2, leaf gas exchange was determined at heading (Z59; Zadoks *et al*.^55^) using a portable infrared gas analyser with a PAM‐Fluorometer (WALZ GFS-3000FL). The cuvette had an area of 4 cm^2^ and measurement conditions were a 700 µmol min^−1^ flow rate, 400 µmol mol^−1^ of reference CO_2_, 24 °C leaf temperature, 1,500 µmol m^−2^ s^−1^ of PPFD and 60–65% relative humidity. Measurements were taken from five flag leaves per genotype and water condition. Chlorophyll content was determined using a portable non-destructive chlorophyll meter (SPAD 502, Minolta).

### Image system and analysis for green area determination (Experiment 2)

Plants were transported by conveyer through an imaging cabinet hosting a RGB camera, with images taken at three angles (0°, 45° and 90°). This procedure was performed every day from the seedling stages until maturity. Image processing was performed using LemnaGrid software from LemnaTec^57^ in the following sequence: 1) nearest neighbour foreground/background colour separation was used to classify pixels. Two sets of colour intensities, corresponding to foreground (target) and to background (non target) were selected. RGB pixels matching selected intensities were mapped onto the image. A search around mapped pixels was performed to identify pixels with similar intensities that might be part of the foreground/background regions. Once the search was performed, the image was converted to binary form, where 1 is the target (plant) and 0 the background (compost, tray, etc.). 2) Morphological techniques were then applied to deal with pixels that were incorrectly classified. First, morphological erosion was applied to remove small and isolated pixel regions that were incorrectly classified as plant. Second, morphological dilation was applied to correct for pixels located in the border of the images that were incorrectly classified as background. 3) A final filter operation used a Region of Interest (ROI) to mark the approximate region occupied by a wheat plant at maturity. Once the images were segmented the green area was calculated.

### Dry matter partitioning, grain yield and agronomic components

In Experiment 1, wheat plants were harvested at maturity, separated into shoot and roots and dried in a fan-forced oven at 60 °C for 48 h. The spikes were counted and threshed manually. The grains were counted using a feed seed counter (ELE International, USA). The evaluated traits were: DW, GY, root:shoot ratio (R:S), harvest index (HI), number of spikes per plant (SP), number of kernels per spike (KS) and per plant (KP), and thousand kernel weight (TKW). DW and spike weight were also measured at harvest in Experiment 2.

### Plant water-use efficiency

Apparent water use (WU) per genotype and water condition was estimated from the water-supplied data. Water supply during irrigation was fully retained by the substrate, therefore there was no water loss from the pots due to drainage. Evaporation from the pot surface was not prevented, it was assumed similar for all genotypes. Plant water use efficiency was calculated as DW/WU. There were four and five replicate pots per genotype and water condition in Experiment 1 and 2, respectively.

### Carbon discrimination in kernels (Experiment 1)

The carbon isotope ratio (^13^C/^12^C) was determined in mature kernels using an elemental analyser (ANCA-SL, PDZ Europa, UK) coupled with an isotope ratio mass spectrometer, at the Laboratory of Applied Physical Chemistry at Ghent University (Belgium). The ^13^C/^12^C ratios were expressed as carbon isotope composition: δ^13^C = (((^13^C/^12^C)_sample_/(^13^C/^12^C)_standard_) − 1), where sample refers to plant material and standard to the laboratory standards that have been calibrated against international standards from Iso-Analytical (Crewe, Cheshire, UK). The precision of δ¹³C analyses was 0.3‰. Further, the carbon isotope discrimination (Δ^13^C) of kernels was calculated as: Δ^13^C (‰) = (δ^13^Ca − δ^13^Cp)/[1 + (δ^13^Cp)/1000], where a and p refer to air and the plant, respectively^58^.

### Data analysis

Differences among genotypes (G) and water condition (W) were determined through analysis of variance (ANOVA) using the general linear model (GLM) procedure of the SPSS statistical package. In addition, relationships between traits were performed using Pearson correlations, and linear and non-linear regression analysis. For the leaf gas exchange, chlorophyll fluorescence and chlorophyll index data, the ANOVAs were performed using G, W and phenology (P) as fixed factors. For the plant traits, the ANOVAs were performed using G and W as fixed factors.

## Supplementary information

Supplementary information.
