# Supplementary material for: Genotypic variations in leaf and whole-plant water use efficiencies are closely related in bread wheat genotypes under well-watered and water-limited conditions during grain filling
Source: Sci Rep. 2020 Jan 16;10:460. doi: 10.1038/s41598-019-57116-0 (PMC6965644; doi:10.1038/s41598-019-57116-0)
Supplement: Supplementary file 1 — Supplementary information. [file 41598_2019_57116_MOESM1_ESM.docx]

**Supplementary Figure 1**. Soil water content from emergence to physiological maturity of wheat plants growing in a glasshouse under well-watered (WW) and water-limited (WL) conditions, in 2015. Commencement of the two water regimes (8 September) was when most of genotypes presented flag fully expanded (Z41). The values are mean of 3 measurements per water regimen.

**Supplementary Figure 2**. Average values (n=5) of water use in six genotypes of spring wheat growing in a Lemnatech glasshouse under well-watered (WW) and water-limited (WL) conditions, in 2015. Beginning of the two water regimes was at 31 days after sowing when most of genotypes presented flag leaf fully expanded (Z41).

**Supplementary Figure 3**. Mean temperature in the glasshouse in Talca, 2015.
